# Western Amazon Basin limits: conservation status and distribution of river dolphins (*Inia geoffrensis* and *Sotalia fluviatilis*) in Ecuador

**DOI:** 10.7717/peerj.21586

**Published:** 2026-07-29

**Authors:** Jessica Pacheco-Esquivel, Santiago Varela, Michelle Vela-Torres, Víctor Utreras, Patricio Macas, Mariana Paschoalini, Hugo Trávez, Ismael Fernandez, Jorge Brito

**Affiliations:** 1 Grupo de Investigación para la Conservación de Delfines de Río, Quito, Ecuador; 2 World Wide Fund for Nature (WWF), Quito, Ecuador; 3 Instituto Nacional de Biodiversidad (INABIO), Quito, Ecuador; 4 Parque Nacional Yasuní, Orellana, Ecuador; 5 World Wide Fund for Nature (WWF), Brasilia, Brazil

**Keywords:** Critically endangered, Distance sampling, Ecuadorian amazon, Freshwater predators, Population assessment, Ramsar wetlands

## Abstract

River dolphins (*Inia geoffrensis* and *Sotalia fluviatilis*) are apex predators in Ecuador’s Amazon, where they face critical endangerment due to their restricted western range limit and escalating anthropogenic pressures. Despite their critical contributions to freshwater ecosystem health, comprehensive population assessments have historically been scarce. Using standardized protocols from the South American River Dolphin Initiative (SARDI), we conducted boat-based surveys spanning 972.6 km across 11 rivers, employing dual-platform distance sampling with habitat stratification. We analyzed 1,141 georeferenced records from Ecuador’s National Biodiversity Database, validated through spatial modeling. Density and abundance were estimated using Distance software with satellite-derived habitat areas. Both species exhibited densities substantially lower than those reported for central Amazonian populations, ranging from 0.33–7.61 ind/km^2^ for *Inia* and 0.05–0.45 ind/km^2^ for *Sotalia*, consistent with their national Critically Endangered status. We document inter-annual fluctuations in key protected areas while updating known species distributions, confirming *Inia* in 37 rivers and recording *Sotalia* in 13 rivers, establishing Ecuador as their westernmost Amazonian range limit. Group sizes varied significantly by habitat, with confluences supporting larger aggregations. This first nationwide assessment reveals acute vulnerability of Ecuador’s river dolphins at their distributional edge. The primary value of this work is providing a standardized baseline against which future surveys can detect population changes under increasing anthropogenic pressures. Urgent implementation of Ecuador’s Action Plan for river dolphin conservation is needed, prioritizing rivers and confluences with confirmed occurrences, transboundary coordination, and community-based monitoring to prevent local extirpations.

## Introduction

The western Amazon basin in Ecuador represents a critical biogeographical boundary for freshwater cetaceans, marking the westernmost limit of their continental distribution (Gómez-Salazar et al., 2012; Utreras, Trujillo & Usma, 2013). This Andean-Amazon transition zone is characterized by hydrographic systems that are narrower and swifter than their central Amazonian counterparts, creating unique ecological conditions that shape population dynamics at the edge of the species’ range (Bernal et al., 2012; Abad et al., 2024). Within this context, Ecuador hosts two river dolphin species, the Amazon River dolphin (*Inia geoffrensis*) and the tucuxi (*Sotalia fluviatilis*) (Utreras, Trujillo & Usma, 2013; Tirira, 2017), which function as apex predators in the region’s freshwater ecosystems, regulating prey populations and maintaining ecological balance (Da Silva et al., 2018a; Da Silva et al., 2018b; Da Silva et al., 2023; Gómez-Salazar et al., 2012).

Freshwater dolphins worldwide face escalating threats due to the high sensitivity of their habitats to anthropogenic disturbances (WWF, 2020; Brum et al., 2021). These cetaceans are characterized by longevity, advanced parental care, and low reproductive rates, making them particularly vulnerable to population declines (Da Silva et al., 2018a; Da Silva et al., 2018b; McGuire & Aliaga-Rossel, 2007). River dolphin habitats across the Amazon basin are increasingly compromised by land-use changes, illegal hunting, hydroelectric dam construction, overfishing, and pollution from hydrocarbon and mineral extraction (Castello & Macedo, 2016; Campbell et al., 2022). Their ecology is intimately tied to pronounced seasonal hydrological cycles (Junk, Bayley & Sparks, 1989; Martin & Da Silva, 2004), which are now being disrupted by climate change-induced extreme floods and droughts (Bodmer et al., 2018; Espinoza et al., 2013). During flood seasons, dolphins access prey in inundated forests (Arraut et al., 2010), while during dry periods they follow fish concentrations toward larger rivers and channels (Junk, Soares & Bayley, 2007; Gómez-Salazar et al., 2012). This environmental heterogeneity creates dynamic prey distribution patterns that fundamentally shape river dolphin spatial ecology across the Amazon’s complex aquatic mosaic (Paschoalini et al., 2021).

Despite their ecological importance and vulnerability, comprehensive population assessments for river dolphins in Ecuador have remained surprisingly scarce. Available data have been collected through localized surveys employing inconsistent methodologies (Utreras, Suárez & Jalil, 2010; Utreras, Trujillo & Usma, 2013; Gómez-Salazar et al., 2012), creating significant spatial, temporal, and methodological gaps in our understanding. Consequently, prior to this study, no standardized, nationwide assessment existed for either species in Ecuador. The country’s small Amazonian territory, constituting merely 1.6% of the total basin (RAISG, 2020), combined with its position at the western distributional edge, has historically relegated Ecuadorian populations to the periphery of research efforts focused on larger, more accessible central Amazonian populations. Consequently, population trends, accurate distribution limits, and density estimates at this critical range margin have remained poorly characterized.

The South American River Dolphin Initiative (SARDI), established in 2006, addressed these methodological inconsistencies by developing standardized regional protocols for population monitoring (Trujillo et al., 2010; Gómez-Salazar et al., 2012). Prior approaches suffered from several critical flaws: variable transect designs prevented cross-study comparisons, inconsistent seasonal sampling introduced temporal bias, and the absence of standardized detection protocols yielded incomparable encounter rates. SARDI’s dual-platform distance sampling methodology, coupled with habitat stratification and rigorous covariate analysis, now enables robust density estimation and population comparisons across the continent (Paschoalini et al., 2021). The 2010–2020 Action Plan for South American River Dolphins explicitly identified the urgent need for reliable density and abundance estimates to assess and monitor population status throughout the region (Trujillo et al., 2010).

The limited available data from Ecuador, combined with mounting evidence of anthropogenic pressures, have led to both species being classified as Critically Endangered (CR) within the country (Utreras, Tirira & Denkinger, 2001a; Tirira, 2021). However, this designation has rested on incomplete information, underscoring the critical need for systematic assessment. The first rigorous study in Ecuador was conducted along the Lagartococha River (Utreras, 1996), but subsequent efforts remained sporadic and geographically restricted, leaving fundamental questions unanswered: What are the current density estimates for dolphins at Ecuador’s western range limit? How are individuals distributed across the country’s diverse river systems? Are the groups occupying Ecuadorian rivers stable, declining, or recovering?

This study addresses these knowledge gaps by providing the first comprehensive, standardized evaluation of the geographic distribution, density, and abundance of *I. geoffrensis* and *S. fluviatilis* across the Ecuadorian Amazon. Through systematic expeditions employing SARDI protocols, we establish robust baseline estimates for these cetaceans at the western edge of their range, complementing previous abundance assessments and enabling improved understanding of population trends. These findings provide a critical foundation for developing effective conservation strategies to safeguard the ecological integrity of Ecuador’s river systems and their emblematic apex predators.

## Materials & Methods

### Ethics statement

All research activities, including field monitoring, were conducted in strict accordance with Ecuadorian wildlife protection laws and approved by the Ministry of Environment, Water, and Ecological Transition (Research Permit no MAATE-ARSFC-2023-3092). Field protocols adhered to international best practices for non-invasive cetacean studies (Gales et al., 2009; Willems et al., 2021), prioritizing minimal disturbance to wild dolphin populations. All personnel completed ethical training in river dolphin research methods prior to data collection, following the capacity-building guidelines of the South American River Dolphin Initiative (SARDI; Trujillo et al., 2010) and the best practices outlined in Willems et al. (2021). This training included instruction on non-invasive observation techniques, distance sampling protocols, and cetacean identification, ensuring consistency with international standards for freshwater cetacean research.

### Study area

**Figure 1:**
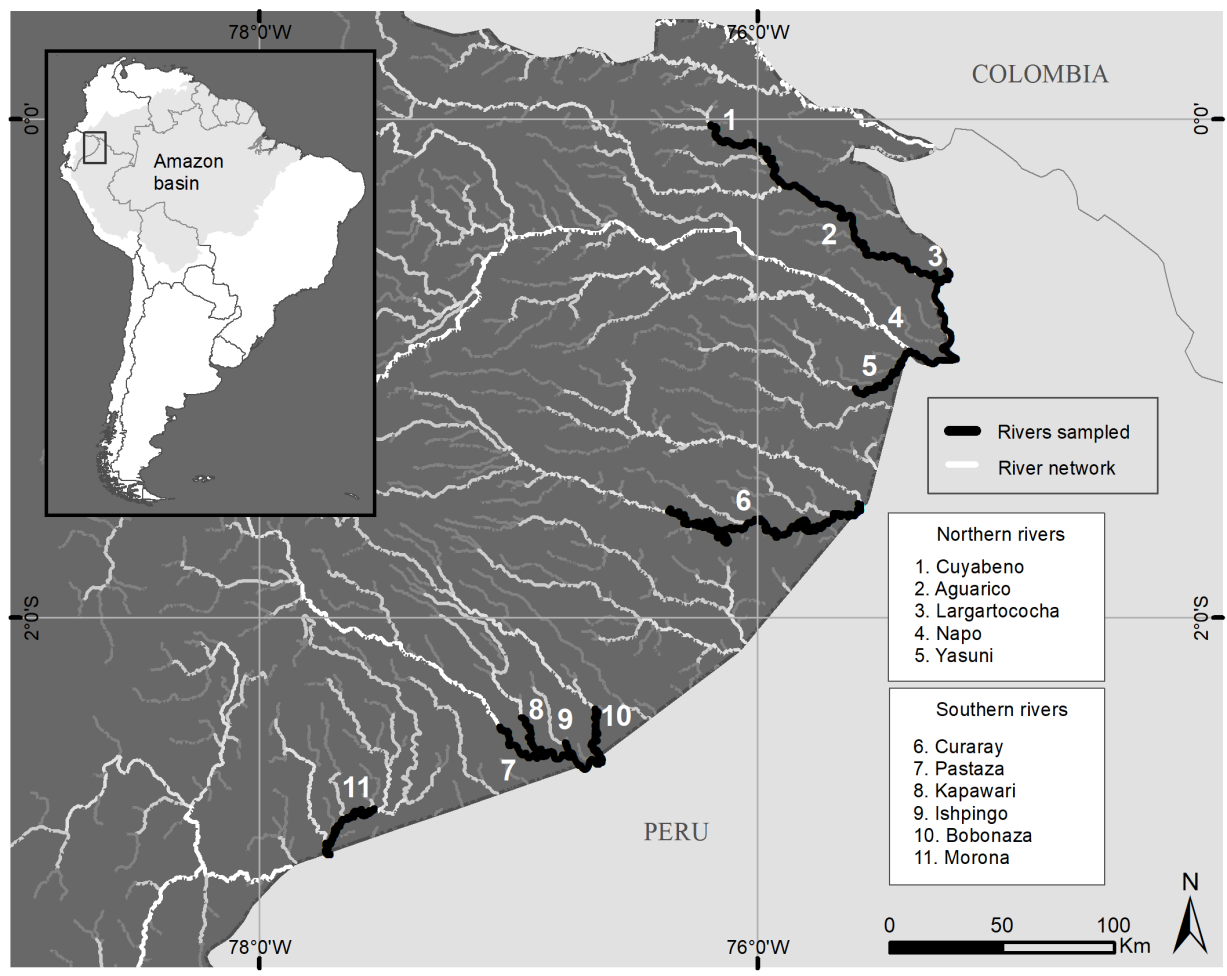
Study area and surveyed rivers in the Ecuadorian Amazon. The inset map shows the location of the study area (black square) within the Amazon Basin (light grey). The main map depicts the river networks (white lines) and the rivers surveyed (black lines) for river dolphin populations between 2019 and 2024.

To assess current population status, we analyzed data from standardized boat-based surveys (2006–2024) covering 972.6 km across 11 rivers in the Ecuadorian Amazon (Fig. 1), sampling 205.87 km^2^ of water surface (Table 1).

**Table 1:** Surveyed rivers, habitat characteristics, and sampling effort for river dolphin monitoring in the Ecuadorian Amazon.

**River**	**Water type**	**Coordinates beginning**	**Coordinates end**	**Number of transects**	**Sampling effort (km)**	**Observation effort (person-hours)**	**Area (km** ^**2**^ **)**
Cuyabeno	Black	−0.014101°S	−0.258948°S	38	94.2	17.1	5.77
		−76.183359°W	−75.899036°W				
Aguarico	White	−0.259813°S	−0.958549°S	49	121.7	22.1	53.58
		−75.900301°W	−75.204050°W				
Lagartocoha	Black	−0.654741°S	−0.463844°S	28	71	12.9	4.58
		−75.261135°W	−75.344191°W				
Napo	White	−0.820603°S	−0.968430°S	27	68	12.4	52.37
		−75.542273°W	−75.198171°W				
Yasuni	Black	−0.931601°S	−0.938251°S	53	132	24	8.62
		−75.387636°W	−75.882566°W				
Curaray	White	−1.576081°S	−1.548308°S	82	205	37.3	29.82
		−76.355376°W	−75.582002°W				
Pastaza	White	−2.439320°S	−2.611428°S	35	86.8	15.8	34.57
		−77.032898°W	−76.633656°W				
Kapawari	Black	−2.546487°S	−2.376069°S	20	50.3	9.1	2.37
		−76.839353°W	−76.946819°W				
Ishpingo	Black	−2.552710°S	−2.502041°S	5	12.5	2.3	0.63
		−76.737790°W	−76.770302°W				
Bobonaza	Black	−2.589989°S	−2.365903°S	25	62.3	11.3	6.16
		−76.633790°W	−76.648550°W				
Morona	White	−2.955017°S	−2.771160°S	28	68.8	12.5	7.4
		−77.713056°W	−77.535186°W				

**Notes.**

Person-hours represent effective sampling effort, calculated based on survey distance, an average vessel speed of 11 km/h (following SARDI protocols), and two active observers per shift due to team rotations. Minor variations reflect local river conditions.

The 11 rivers were selected using a stratified approach based on three criteria: (1) representativeness of the region’s hydrographic diversity, including both white-water rivers of Andean origin (Aguarico, Napo, Curaray, Pastaza, Morona) and black-water rivers of forest origin (Cuyabeno, Lagartococha, Yasuni, Kapawari, Ishpingo, Bobonaza) to capture the full spectrum of habitats available to dolphins; (2) accessibility during both rising- and falling-water seasons, ensuring that surveys could be consistently replicated across hydrological periods; and (3) conservation priority, prioritizing rivers within or adjacent to protected areas (Cuyabeno Wildlife Reserve, Yasuni National Park, Cuyabeno–Lagartococha–Yasuni Ramsar site (hereafter CLY)) and those identified as data-deficient in previous assessments (*e.g.*, Curaray, Morona, Pastaza tributaries). This design ensures that our sample encompasses the ecological variability of Ecuador’s Amazonian watersheds while targeting areas of highest conservation relevance.

### Field data collection

**Figure 2:**
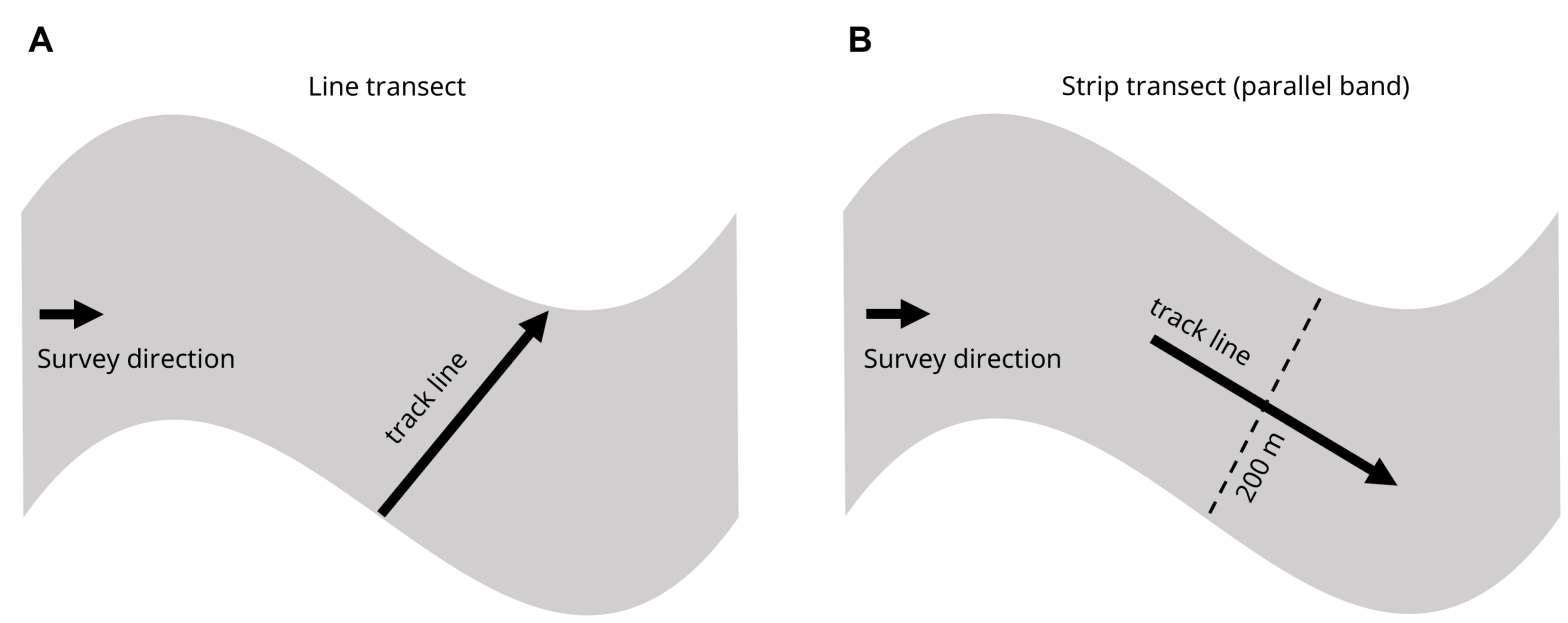
Schematic representation of survey designs used during river dolphin monitoring. (A) Line transects follow a shore-to-shore navigation track crossing the full river channel, with observers recording detections and perpendicular distances. (B) Strip transects run parallel to shore in narrow tributaries, recording detections within a fixed 200 m band.

We conducted visual surveys from boats using a combination of cross-channel (line transects for major rivers) and strip transects (for tributaries) (Fig. 2), following the standardized methodology developed by the South American River Dolphin Initiative (SARDI) and described in Gómez-Salazar, Trujillo & Whitehead (2012) and Williams et al. (2016).

Each transect had a standard length of 2.5 km. For linear transects, vessels navigated along the river’s midline, maintaining a constant distance from each bank. Strip transects were conducted parallel to the river margins, maintaining an average distance of 200 m from the shoreline. Surveys were conducted during both rising- and falling-water seasons (transitional periods) to standardize sampling across the hydrological cycle, following recommendations from Williams et al. (2016) for robust long-term population monitoring. This strategic choice prioritizes a multi-year framework that captures the full spectrum of habitat use and dolphin distribution patterns across hydrological phases, rather than optimizing short-term detectability during a single season.

For both transect types, we implemented the following standardized observation protocols:

1. Dual-platform observer system

Two observation platforms (bow and stern) were installed on each boat, each staffed by at least two observers and one data recorder, with team members rotating hourly between three positions (port-side observer, data recorder, and starboard-side observer) while replacing active observers with rested team members; platforms maintained continuous communication to maximize detection accuracy and minimize duplicate counts (Paschoalini et al., 2021).

2. Standardized observation conditions

Standardized observation protocols required maintaining an observation height of ≥2.5 m above the water surface, which was consistent across all surveys as the same boat types were used throughout all expeditions in Ecuador. This uniformity eliminates potential variability in detectability associated with observer height; therefore, this factor was not included as a covariate in detection function models. Vessels traveled at a constant speed of 10–12 km/h to minimize behavioral disturbance during surveys.

3. Data recording parameters

For each sighting, observers documented: species identification, group size (including calves), radial distance (laser rangefinder, Nikon Prostaff 1,000), sighting angle (angle boards), distance to nearest river margin, habitat type (main river, tributary, confluence, lake, channel, or island; Table 2), riverbank type for strip transects (human-modified shoreline, beach, flooded forest, pasture, terra firme, ravine, or rock), and visibility conditions (low, moderate, good, or optimal). To minimize estimation errors, all distance measurements were instrument-based, observers were trained in rangefinder and angle board protocols, and bow and stern platforms recorded measurements independently for cross-validation. Sightings from each platform were treated as independent events; a stern-platform sighting was classified as new (not a duplicate) based on species, group size consistency, and similarity in radial angles and distances at initial detection (Paschoalini et al., 2021).

**Table 2:** Description of aquatic habitat types surveyed for river dolphins in the Ecuadorian Amazon, based on criteria from Trujillo et al. (2010), Gómez-Salazar et al. (2012) and Aliaga-Rossel & Duran (2020).

**Type of habitat**	**Characteristics**
Main river	Andean-origin rivers are characterized by white, dark brown, or yellowish-brown waters with low transparency, resulting from high suspended sediment loads. In Ecuador, these rivers typically exceed 400 m in width, exemplified by the Napo and Aguarico rivers
Tributaries	Small-to-medium rivers (width ≤400 m) characterized by dark or clear waters of forest origin, such as the Cuyabeno and Yasuní rivers.
Confluence	Zones between main rivers and tributaries maintain perennial connectivity across all hydrological seasons. Typically exhibit water mixing (white waters with dark/clear waters). Examples include the Aguarico-Lagartococha confluence.
Lake	Waterbodies of variable size surrounded by land, characterized by distinct physicochemical properties (e.g., water color, pH, transparency) and geomorphology, representing unique habitat types and ecosystems. Some form through isolation of former river channels over time.
River island	Vegetated landforms within river channels that emerge and disappear in response to hydrological dynamics.

**Notes.**

The ‘River island’ category includes transient islands shaped by seasonal sediment deposition and erosion.

4. Environmental data collection

Environmental parameters were recorded at the start and end of each transect, as well as during every sighting event (including habitat type, riverbank characteristics, water type, and visibility conditions). Each study also documented the annual flood pulse cycle: low-water (dry season), high-water (wet season), and transitional periods (Junk, Bayley & Sparks, 1989).

### Analysis

Density, encounter rates, and abundance were estimated separately for each river using a stratified design (Table 2). Data were analyzed in R (v4.1.3; R Core Team, 2015) with the Distance (Miller, 2017) and mrds (Laake et al., 2017) packages, implementing distance sampling (DS) methods (Buckland et al., 2001) to calculate densities/abundances for mid-river sections.

Density for line transect data was derived using the Horvitz-Thompson estimator (Horvitz & Thompson, 1952) for each species as follows:

\begin{eqnarray*}\hat {D}= \frac{E(s)}{2{w}_{i}{L}_{i}} \sum _{i=1}^{n} \frac{1}{g(x,z)} \end{eqnarray*}

where *E(s)* = expected group size, *w* = maximum width by stratum, *L* = transect length by stratum, and *n* = number of detected groups. Detection probability was modeled using multiple covariate distance sampling (MCDS) as *g* (*x*, *z*), where *x* is perpendicular distance and *z* represents covariates affecting detectability (Marques & Buckland, 2003). Candidate covariates, group size (gs), platform (pt), species (sp), and visibility (sg), were tested individually. The best-fitting model was selected using Akaike’s Information Criterion (AIC; Buckland et al., 2001).

Strip transects (200 m width) were employed in narrow tributaries characteristic of Ecuadorian headwaters, where reduced channel width precluded conventional distance sampling. This adaptation enabled systematic coverage of aquatic habitats in inaccessible areas, ensuring reliable data collection across diverse hydrologic and geomorphic conditions. Parallel transect data were analyzed with habitat stratification (main river, tributary, confluence, lake, and island) following Gómez-Salazar et al. (2012). Strip-transect density was calculated as:

\begin{eqnarray*}{D}_{i}= \frac{{E}_{i} \left[ \frac{{n}_{0-50}}{P2} + \frac{{n}_{50-100}}{P1} + \frac{{n}_{100-150}}{P1} + \frac{{n}_{150-200}}{P2} \right] }{W{L}_{i}g(0)} ,\mathrm{for}i=1,\ldots ,n \end{eqnarray*}

where D_i_ represents density in habitat i, E_i_ is the estimated group size in habitat i, L_i_ denotes total transect length in habitat i, N is the number of habitats studied, W indicates strip width (200 m), and g (0) reflects detection probability at zero distance, calculated as (1 −q^2^) with q being the equal probability of missing dolphin groups on bow/stern platforms (assuming independent sightings). Correction factors P_1_ and P_2_ accounted for undetected groups per 50 m band (Frias, 2019), with species-specific values: for *I. geoffrensis*, P_1_ = 0.960 and P_2_ = 0.630 (shape = 0.37 ± 0.12, scale = −2.61 ± 0.42); for *S. fluviatilis*, P_1_ = 0.998 and P_2_ = 0.893 (shape = 0.99 ± 0.15, scale = −2.24 ± 0.41).

Encounter rates (ER) were calculated as a relative abundance index for each river and survey campaign using:

\begin{eqnarray*}ER= \frac{\sum {s}_{i}}{L} \end{eqnarray*}

where ∑*s*_*i*_ is the total number of individuals detected and *L* is the total transect length surveyed (km). This metric provides a standardized measure of dolphin occurrence proportional to density under comparable conditions (Gómez-Salazar et al., 2012). Encounter rates and their 95% confidence intervals were estimated per survey period and site based on detections per kilometer. As encounter rates are not corrected for detection probability, they should be interpreted as relative indices rather than absolute abundance measures (Buckland et al., 2001). Nonetheless, ER offers valuable complementary information, particularly when sample sizes are too low for robust density estimation, by enabling detection of spatial or temporal patterns despite high uncertainty.

#### Confidence interval calculation

For encounter rates, 95% confidence intervals were calculated as:

\begin{eqnarray*}95\%~CI=ER~\pm ~(t~\times ~SE), \end{eqnarray*}

where *SE* is the standard error of the encounter rate, derived from variation in encounter rates among transects within each survey (*SE* = *SD*/√*n*), and *t* is the critical value from the t-distribution with *n–1* degrees of freedom (Zar, 2010).

For density estimates, 95% confidence intervals were derived from the coefficient of variation (CV) using a log-normal approximation (Buckland et al., 2001; Gómez-Salazar et al., 2012):

\begin{eqnarray*}95\%~CI=D\times \exp \nolimits \left( \pm 1.96\times \sqrt{\ln \nolimits (1+C{V}^{2})} \right) . \end{eqnarray*}

This approach assumes log-normality of the density estimator and propagates uncertainty from detection probability, encounter rate variance, and habitat-specific area estimates (Buckland et al., 2001).

Total density (D) for both species across each river study area was calculated as the weighted mean of estimated abundance (summed across habitat types) divided by the total area (km^2^). Variances followed Gómez-Salazar et al. (2012) methods, with global CV computed as:

\begin{eqnarray*}\mathrm{CV}= \frac{\sqrt{\sum (SE_{i}^{2})}}{\sum Di} \end{eqnarray*}

where *SE*i is the standard error of the density in habitat i.

General abundances (N) in each river for each species were obtained by summing the estimated abundances (*D*i ×*A*i) in each habitat type (i) using:

\begin{eqnarray*}N=\sum _{i=1}^{n}{D}_{i}{A}_{i},~~~\mathrm{for}~i=1,\ldots ,n \end{eqnarray*}

where *n* is the number of surveyed habitats and A_i_ represents the study area (km^2^) for habitat i, calculated using Sentinel-2 satellite imagery from dates proximate to field surveys *via* Google Earth Engine’s open-access repository. A_i_ values were computed per campaign and per river. Image processing and refinement were performed using ArcGIS (version 10.3). For accurate area calculations, we integrated field-collected data consisting of: (1) GPS coordinates marking transect start and end points, and (2) channel width measurements acquired with laser rangefinders at 2.5 km intervals along each river.

This study focused on estimating overall river densities and abundances rather than habitat-specific densities, adopting a national-scale approach to assess population variability across Ecuador’s major rivers and tributaries.

### Occurrence data collection and processing

#### Distribution range analysis

To update the distribution ranges and river-specific occurrence patterns of *Inia geoffrensis* and *Sotalia fluviatilis* in Ecuador, we analyzed 1,061 and 80 georeferenced records for each species, compiled from the National Biodiversity Database (Instituto Nacional de Biodiversidad, 2025) for the period 1996–2024. This multi-source repository integrates data from researchers, conservation programs, and global platforms. These records complement standardized SARDI survey data by providing historical context and extending geographic coverage to rivers not yet surveyed with boat-based protocols. Records were filtered to elevations < 300 m based on validated species thresholds (Utreras, Trujillo & Usma, 2013; Tirira, 2017; MAATE, 2025).

#### Data processing protocol

All occurrence records underwent a two-stage quality control process. First, we removed duplicate entries using Microsoft Excel’s Remove Duplicates tool (Microsoft Corporation, 2023), matching both geographic coordinates and observation dates. Second, we performed spatial validation in ArcGIS 10.3 (ESRI, 2014) by projecting all records onto official Ecuadorian hydrographic networks (Ministry of the Environment, Water and Ecological Transition of Ecuador, MAATE)(2015). Following established georeferencing best practices (Chapman & Wieczorek, 2020), we excluded records located >500 m from mapped waterways and visually verified the remaining points against high-resolution satellite imagery to ensure accurate riverine alignment.

## Results

The distribution of Amazon River Dolphin (*Inia geoffrensis*) populations across Ecuadorian rivers demonstrates complex spatial organization and temporal dynamics. Our integrated analysis of density estimates and encounter rates (Table 3) reveals marked heterogeneity across basins, with some rivers supporting persistently low densities while others exhibit notably high values or extreme inter-annual fluctuation. For *Sotalia fluviatilis*, population estimates were obtained exclusively from Ecuador’s northern Amazon rivers (Table 4), where systematic surveys have confirmed characteristically low densities and highly variable encounter rates, reinforcing the species’ reputation for rarity and discontinuous distribution at its western range limit (Denkinger, 2010; Utreras, Tirira & Denkinger, 2001b; Zapata-Ríos & Utreras, 2004; Gómez-Salazar et al., 2012).

**Table 3:** Encounter rates, density estimates, and temporal variability of Inia geoffrensis across Ecuadorian river systems (2006–2024).

**River**	**Effort (km)**	**Obs**	***N***	**ER (ind/km) ± 95% ME**	**D (Ind/km**^**2**^) **(95% CI)**	**CV**	**Year**	**Data source**
Cuyabeno	87.5	9	22	0.25 (± 0.14)	4.19 (1.39–12.62)	0.6	2019	WWF (2019) ^a^
Cuyabeno	167	15	34	0.20 (± 0.05)	3.32 (2.57–4.28)	0.1	2020	Utreras & Sarmiento (2020)
Cuyabeno	170	10	23	0.14 (± 0.04)	2.21 (1.62–3.02)	0.2	2023	WWF (2023) ^a^
Cuyabeno	94.2	12	16	0.17 (± 0.05)	2.42 (1.84–3.18)	0.1	2024	WWF (2024) ^a^
Aguarico	147.5	17	33	0.22 (± 0.09)	0.66 (0.52–0.83)	0.1	2019	WWF (2019) ^a^
Aguarico	51.03	12	20	0.39 (± 0.13)	1.49 (0.54–4.15)	0.6	2021	WWF (2021) ^a^
Aguarico	121.7	6	13	0.11 (± 0.06)	0.31 (0.24–0.40)	0.1	2024	WWF (2024) ^a^
Lagartococha	17.5	6	15	0.86 (± 0.75)	6.53 (2.17–19.66)	0.6	2019	WWF (2019) ^a^
Lagartococha	23	6	8	0.35 (± 0.14)	3.07 (1.55–6.09)	0.4	2021	WWF (2021) ^a^
Lagartococha	55	15	27	0.49 (± 0.09)	7.61 (5.90–9.81)	0.1	2022	Omacha Foundation (2022)
Lagartococha	15	8	11	0.73 (± 0.24)	3.01 (2.29–3.96)	0.1	2024	WWF (2024) ^a^
Ramsar CLY^**^	196.9	27	147	0.75	3.53 (2.47–3.13)	0.1	2006	Gómez-Salazar et al. (2012)
Napo^*^	62.02	5	7	0.11 (± 0.05)	1.59 (0.11–22.47)	2.3	2021	WWF (2021) ^a^
Yasuní	145	17	47	0.32 (± 0.09)	4.96 (3.84–6.39)	0.1	2020	Utreras & Sarmiento (2020)
Yasuní	71.11	15	19	0.27 (± 0.05)	2.22 (0.21–23.09)	1.8	2021	WWF (2021) ^a^
Yasuní	132	18	26	0.19 (± 0.05)	3.02 (2.43–3.75)	0.1	2023	Macas P., Parque Nacional Yasuní
Yasuní	81	7	9	0.11 (± 0.04)	2.03 (1.43–2.88)	0.2	2024	WWF (2024) ^a^
Curaray	205	9	12	0.06 (± 0.02)	0.40 (0.29–0.55)	0.2	2023	WWF, Expedición Curaray^a^
Pastaza	86.8	7	8	0.21 (± 0.03)	0.53 (0.37–0.75)	0.2	2022	WWF (2022) ^a^
Pastaza	86.8	3	6	0.15 (± 0.05)	0.33 (0.03–3.29)	1.7	2024	WWF, Expedición Pastaza^a^
Kapawari	36.85	3	4	0.11 (± 0.01)	2.72 (1.59–4.66)	0.3	2022	WWF (2022) ^a^
Kapawari	50.3	4	7	0.14 (± 0.02)	3.48 (2.19–5.53)	0.2	2024	WWF, Expedición Pastaza^a^
Bobonaza	53.4	6	7	0.13 (± 0.01)	1.54 (1.09–2.19)	0.2	2022	WWF (2022) ^a^
Bobonaza	62.3	5	8	0.13 (± 0.02)	1.62 (1.06–2.48)	0.2	2024	WWF, Expedición Pastaza^a^
Morona	68.8	15	20	0.29 (± 0.03)	2.90 (2.25–3.74)	0.1	2024	WWF, Expedición Morona^a^

**Notes.**

Acronyms Obs Sightings N Number of individuals ER Encounter rate ME Margin of error D Density CI Confidence interval CV Coefficient of Variation

* Transboundary data collected from Ecuador and Peru.

** Data from the CLY Ramsar site includes aggregated records from five river systems.

a Original data collected and analyzed by the authors.

For estimated densities, high CVs and missing values reflect poor data robustness; trends are speculative without more replicates.

**Table 4:** Review of abundance, encounter rate, and density estimates of Sotalia fluviatilis in Ecuadorian rivers (2006–2024).

**River**	**Effort (km)**	**Obs**	***N***	**ER (Ind/km) ± 95% ME**	**D (Ind/km**^**2**^) ** (95% CI)**	**CV**	**Year**	**Data source**
Aguarico	147.5	3	4	0.03 (± 0.03)	0.08 (0.04–0.15)	0.34	2019	WWF (2019) ^a^
Aguarico	51.03	1	1	0.02	0.05 (—)	–	2021	WWF (2021) ^a^
Aguarico	50	2	4	0.08 (± 0.04)	0.18 (0.10–0.30)	0.28	2022	Omacha Foundation (2022)
Ramsar CLY^**^	196.9	5	19	0.09	1.13 (0.21–6.09)	1.37	2006	Gómez-Salazar et al. (2012)
Napo^*^	62.02	2	6	0.09 (± 0.04)	0.45 (0.02–11.93)	2.05	2021	WWF (2021) ^a^
Yasuní	81	1	2	0.02 (± 0.11)	0.38 (—)	–	2024	WWF (2024) ^a^

**Notes.**

Acronyms Obs Sightings N Number of individuals ER Encounter rate ME Margin of error D Density CI Confidence interval CV Coefficient of Variation

* Transboundary data collected from Ecuador and Peru.

** Data from the CLY Ramsar site includes aggregated records from five river systems.

a Original data collected and analyzed by the authors.

CVs based on <10 sightings should be interpreted with extreme caution; they often underrepresent true uncertainty.

### Density distribution patterns

**Figure 3:**
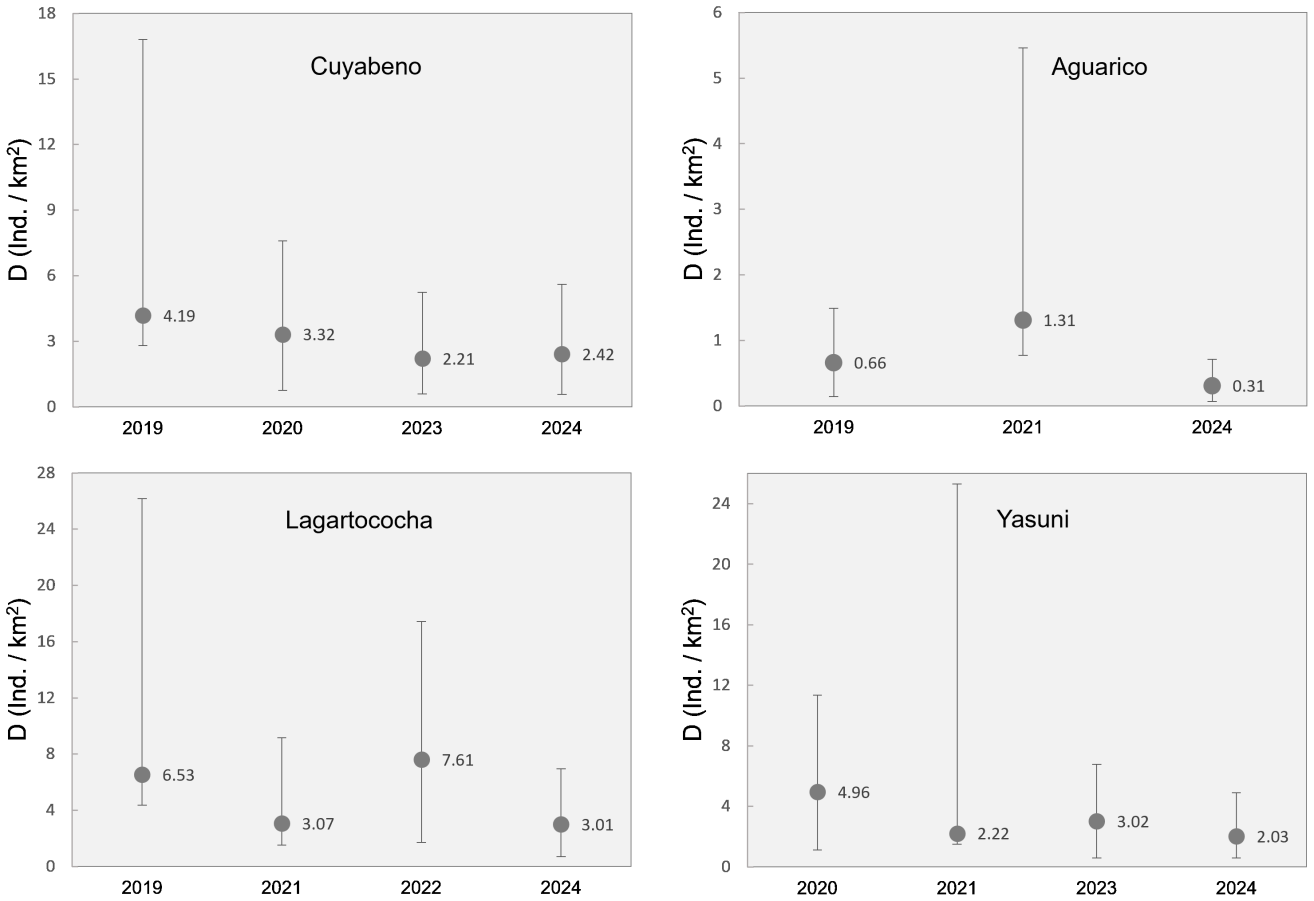
Temporal trends in densities (Ind/km^2^) of *Inia geoffrensis* from four rivers of the CLY Ramsar site (2019–2024). Error bars indicate 95% confidence intervals.

For *Inia geoffrensis*, the highest densities among all surveyed Ecuadorian rivers were recorded in the Lagartococha River, peaking at 7.61 ind/km^2^ (CV = 0.1) in 2022 during the Amazon Perpetual Planet Expedition, followed by 6.53 ind/km^2^ (CV = 0.6) in 2019 (Table 3). However, subsequent surveys revealed considerable fluctuation and inter-annual variability even within this high-density system (3.01 ind/km^2^ in 2024, CV = 0.1). The Cuyabeno and Yasuní rivers also supported densities that were intermediate to high relative to the study-wide range (0.33–7.61 ind/km^2^), ranging from 2.21 to 4.19 ind/km^2^ and 2.03 to 4.96 ind/km^2^, respectively, with generally low coefficients of variation (CV ≤ 0.2) in recent years (Fig. 3). In contrast, densities in several southern and central basins were substantially lower than those in the northern CLY rivers. The Pastaza River system exhibited the lowest densities among all surveyed rivers, with densities ranging from 0.33 to 0.53 ind/km^2^ (2022–2024), while the Curaray River, surveyed in 2023, recorded 0.40 ind/km^2^ (CV = 0.2), among the lowest documented for the species in Ecuador. The Morona River revealed a contrasting pattern, despite its southern location, it supported a density of 2.90 ind/km^2^ (CV = 0.1), which falls within the upper half of the study-wide range (median = 2.42 ind/km^2^). The Ramsar CLY site, aggregating data from five river systems in 2006, yielded a composite density of 2.78 ind/km^2^, but with exceptionally high uncertainty (CV = 3.11).

For *Sotalia fluviatilis*, density estimates were obtained exclusively from Ecuador’s northern Amazon rivers (Aguarico, Napo, and Yasuní) where systematic surveys have been conducted (Table 4). Densities ranged from 0.05 to 0.45 ind/km^2^, which are 1–2 orders of magnitude below central Amazonian values (*e.g.*, 17.14 ind/km^2^; Paschoalini et al., 2021). The sole exception was the aggregated Ramsar CLY estimate from 2006 (1.13 ind/km^2^, CV = 1.4). Coefficients of variation were generally high (*e.g.*, CV = 2.1 for the Napo River in 2021) or unavailable, reflecting small sample sizes and the species’ elusive, patchy distribution.

### Relative abundance (encounter rate) patterns

**Figure 4:**
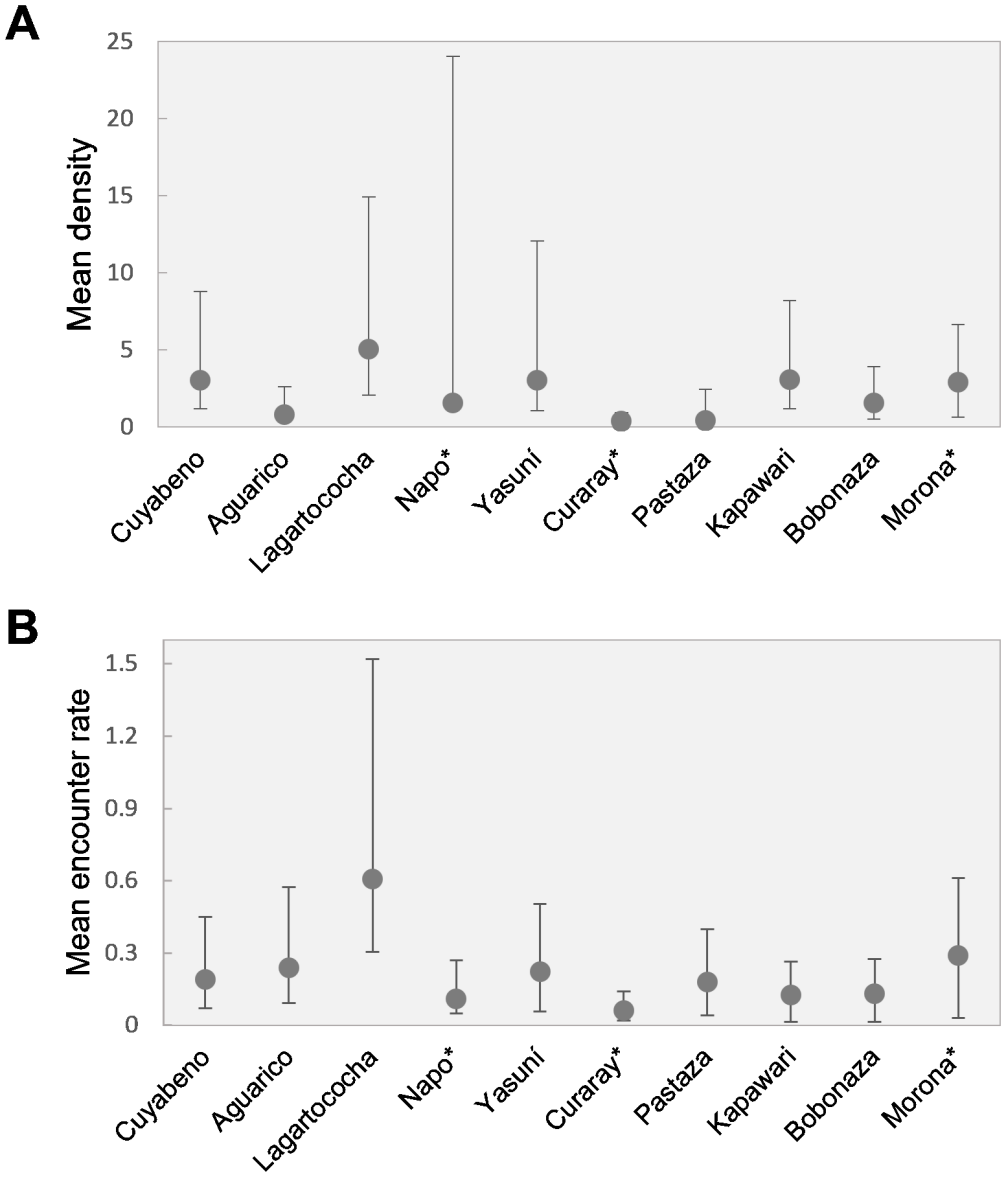
(A–B) Mean density (ind/km^2^) and encounter rate (ind/km) of *Inia geoffrensis* across 10 Ecuadorian rivers (2019–2024). Error bars indicate 95% confidence intervals. Rivers marked with an asterisk (*) represent single surveys.

Similar to density patterns, the encounter rates (individuals/km) for *Inia* exhibited spatiotemporal variation (Table 3). Among all surveyed rivers, the highest rates were consistently documented in Lagartococha River, ranging from 0.49 ind/km in 2022 to 0.73 ind/km in 2024, reflecting this system’s exceptional importance for the species in Ecuador. However, marked inter-annual fluctuation was evident, with rates dropping to 0.22 ind/km in 2021, consistent with the high variability observed in density estimates for this river. Intermediate encounter rates, falling between the high rates of Lagartococha and the low rates of Curaray, characterized several northern and central systems. The Cuyabeno River showed relatively stable rates between 0.14 and 0.25 ind/km across four survey years (2019–2024), while the Yasuní River showed lower values in later years (0.32 ind/km in 2020 *vs.* 0.11 in 2024), but overlapping confidence intervals indicate no statistically significant trend. The Aguarico River followed a similar pattern, dropping from 0.22–0.25 ind/km (2019–2021) to 0.11 ind/km in 2024 (Table 3). In the Pastaza River system, encounter rates (0.15–0.21 ind/km) were similar to the study-wide median of 0.17 ind/km but showed inter-annual variability (2022–2024), though with high uncertainty in 2024 (CV = 1.7). Its tributaries showed lower values: Kapawari ranged from 0.11 to 0.14 ind/km, while Bobonaza maintained stable but low rates (0.13 ind/km) across both survey years. The lowest encounter rates among all surveyed rivers were recorded in the Curaray River (0.06 ind/km in 2023), reinforcing its characterization as a low-density system. The Napo River, based on transboundary data from 2021, showed moderate but highly uncertain rates (0.26 ind/km, 95% CI = ±0.38), reflecting both sampling challenges and the inherent variability of peripheral populations (Fig. 4).

For *Sotalia*, encounter rates (0.02–0.09 ind/km) were an order of magnitude lower than those reported for central Amazonian rivers (*e.g.*, 0.5–1.2 ind/km; Gómez-Salazar et al., 2012) (Table 4). The highest rates occurred in the Napo River in 2021 (0.09 ind/km, CV = 2.1) and the Aguarico River in 2022 (0.08 ind/km, CV = 0.3), though both estimates carry substantial uncertainty. In the Yasuní River, encounter rates dropped to 0.02 ind/km in 2024, consistent with the species’ extreme rarity in southern portions of its Ecuadorian range.

### Group sizes

**Figure 5:**
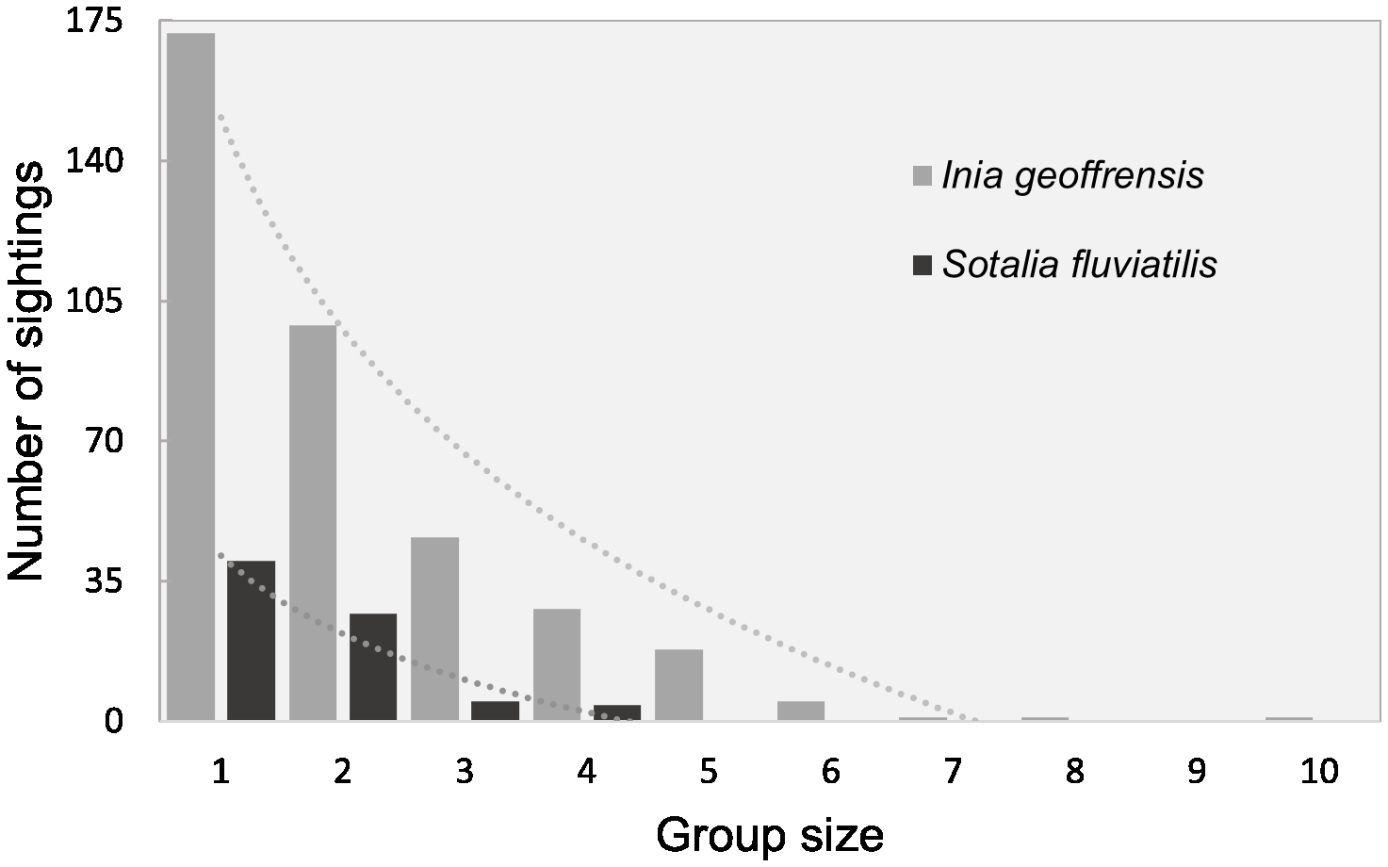
Sightings per group size for *I. geoffrensis* and *S. fluviatilis* in Ecuadorian Amazon.

**Figure 6:**
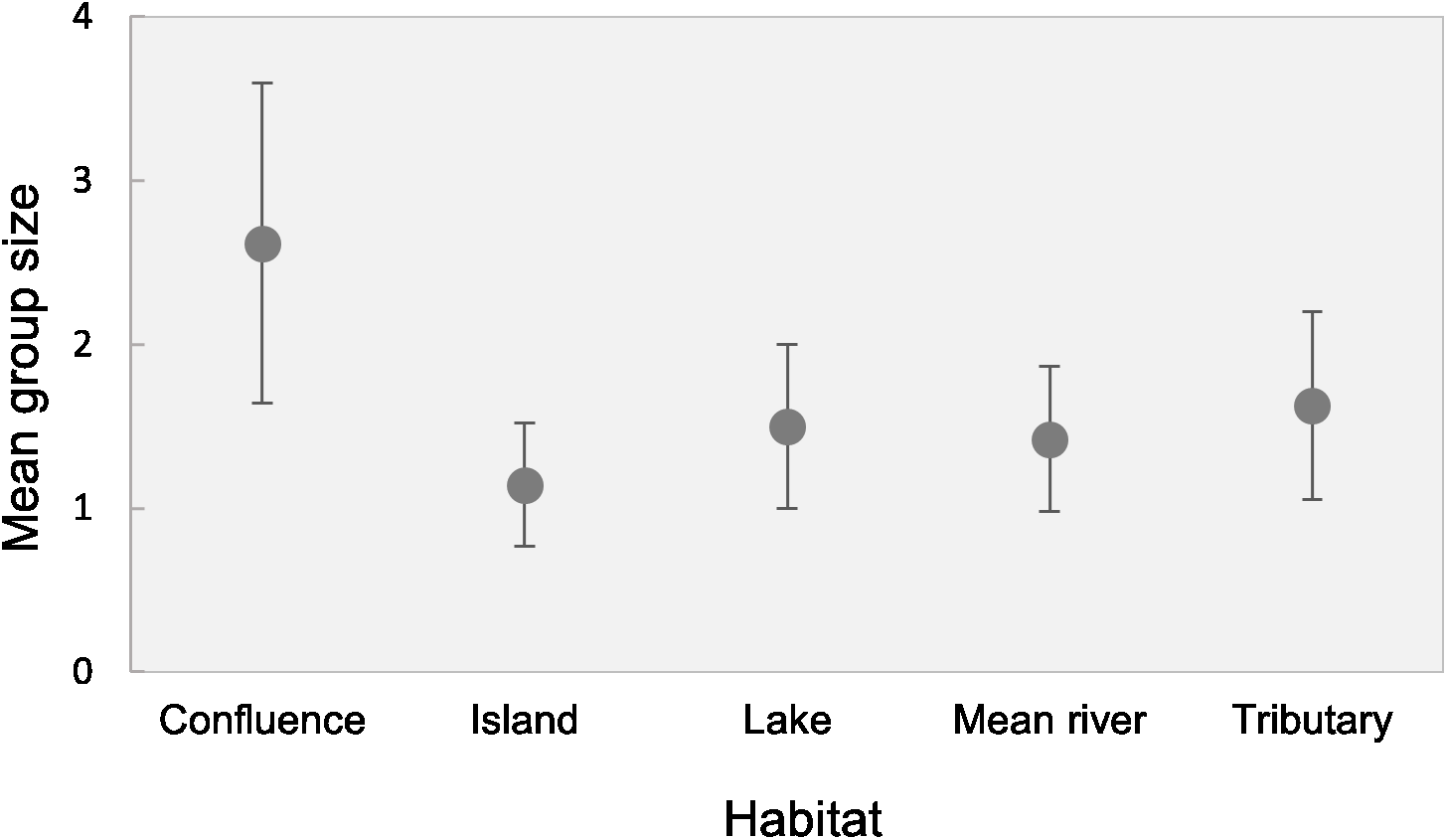
Mean group size and (±*SD*) of *I. geoffrensis* (2006-2024) across habitat types in Ecuadorian Amazon.

The mean group size of *I. geoffrensis* was 1.47 individuals (± 0.32 SD), with a range from 1 to 10 (Fig. 5). Solitary individuals were most frequently observed, comprising 47% of all sightings (n >170), followed by pairs (27%). The largest groups, consisting of 8 and 10 individuals, were each observed once in the Lagartococha River in 1996. All group size data in Table 5 and Figs. 5 and 6 is based on recorded sightings. For *Sotalia fluviatilis*, the mean group size was slightly larger (1.57 ± 0.79 SD), with a range of one (solitary) to four individuals (Fig. 5). Solitary individuals were most frequent (52% of sightings; *n* = 40). While both species exhibited similar mean group sizes, (*I. geoffrensis:* 1.47 ± 0.32; *S. fluviatilis*: 1.57 ± 0.79), *S. fluviatilis* displayed more than twice the variability (SD = 0.79 *vs.* 0.32). Our habitat-specific analyses support this: confluences showed the largest mean group size (2.62 ± 0.98 individuals; CV = 0.37) among all habitat types, indicating moderate dispersion. In contrast, island habitats had the smallest mean group size (1.14 ± 0.38), with variability approximately one-third that of confluences (CV = 0.33). Lakes averaged 1.5 ± 0.5 individuals, while main rivers and tributaries showed intermediate sizes (1.42 ± 0.44 and 1.63 ± 0.57, respectively; Table 5, Fig. 6).

### Distribution range and river-specific occurrence

Our analysis of 1,061 occurrence records for *Inia geoffrensis* and 80 for *Sotalia fluviatilis* from Ecuador’s National Biodiversity Database (Instituto Nacional de Biodiversidad, 2025) reveals updates to both species’ distribution ranges in the Amazon basin.

*I. geoffrensis* occurs in 37 rivers and lacustrine systems across Ecuador’s Amazon basin (<260 m elevation), including the Putumayo, Napo, Tigre, Pastaza, and Morona River basins and their tributaries (Herman, Von Fersen & Solangi, 1996; Utreras, 1996; Utreras, Suárez & Jalil, 2010; Utreras, Trujillo & Usma, 2013; Trujillo et al., 2016; Tirira, 2017; Aguilar & Goucher, 2024; MAATE, 2025).

**Table 5:** Mean group size *E* (s) with standard deviation *SD* (s) and coefficient of variation (*CV*), number of sightings (*n*) of *I. geoffrensis* across habitat types (2006–2024).

**Habitat**	***E (s)***	***SD (s)***	***CV***	***n***
Confluence	2.62	0.98	0.37	21
Island	1.14	0.38	0.33	2
Lake	1.50	0.50	0.33	5
Mean river	1.42	0.44	0.31	32
Tributary	1.63	0.57	0.35	83
Overall^*^	1.66	0.57	0.34	143

**Notes.**

* The overall estimates of E(s), SD(s) and (CV) correspond to the mean among habitats.

**Figure 7:**
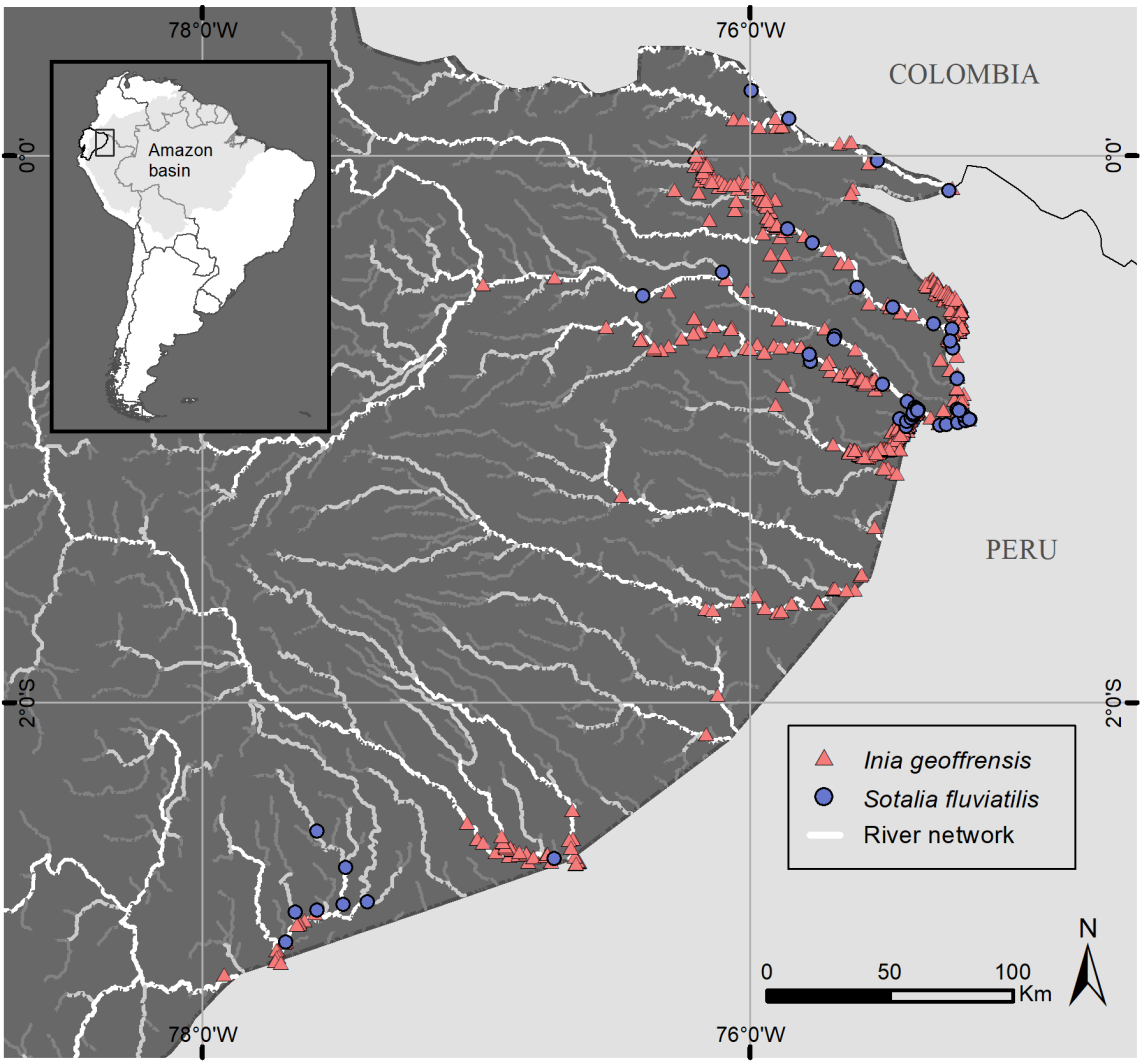
Distribution of *Inia geoffrensis* (red triangles) and *Sotalia fluviatilis* (blue circles) in Ecuador. Occurrences are based on georeferenced records from 1996 to 2024. The river network is shown in white.

Confirmed distribution records since the 1990s document *I. geoffrensis* in five key areas: (1) Putumayo basin (main river, San Miguel, Güeppí); (2) Aguarico system (Cuyabeno, Sábalo, Pacuyacu, Lagartococha, Yanayacu, Cocaya); (3) Napo watershed (Payamino, Coca, Indillama, Pañayacu, Tiputini, Tivacuno, Yasuní, Nashiño, Lobo, Shiripuno, Tiwino, Cononaco, Curaray); (4) southern drainages (Pindoyacu, Conambo, Tigre); and (5) southeastern basins (Pastaza: Kapawari, Ishpingo, Bobonaza; Morona: Wichimi, Makuma, Cangaime, Cushimi, Mangosiza). In addition, the species has been recorded in the lower basin of Santiago River (Fig. 7).

In contrast, *Sotalia fluviatilis* exhibits a restricted distribution in Ecuador’s Amazon basin (<240 m elevation), occurring in 13 rivers across the Putumayo, Napo, Pastaza, and Morona basins. Historical records confirm its presence in the Morona system (Wichimi, Macuma, Cangaime, Cushimi, Mangosiza) and lower Pastaza (Zapata-Ríos & Utreras, 2004; Utreras, Suárez & Jalil, 2010; Utreras, Trujillo & Usma, 2013; Aguilar & Goucher, 2024).

Northern populations persist in the Putumayo (main river and Güeppi tributary) and Aguarico (Cuyabeno confluence to Napo junction). In the Napo watershed, sightings occur from the Tiputini confluence downstream to Peru, with sporadic reports in lower Tiputini and including recent records in the Yasuní-Napo confluence. Southern distribution remains patchy, with occasional records in lower Pastaza and Morona tributaries (Fig. 7).

## Discussion

### Population status at the western range edge and amazonian context

This study provides the first standardized, nationwide assessment of river dolphins in Ecuador, revealing densities for both species that are low relative to central Amazonian benchmarks (Paschoalini et al., 2021) and among the lowest documented across the species’ range (Gómez-Salazar et al., 2012). In Ecuador, the national Red List (Tirira, 2021) classifies both species as Critically Endangered based on thresholds for small population size (<250 mature individuals for *Inia*, <100 for *Sotalia*), restricted area of occupancy (<10 km^2^), and documented continuing decline. Our estimates (*I. geoffrensis*: 0.33–7.61 ind/km^2^; *S. fluviatilis*: 0.05–0.45 ind/km^2^) are consistent with these criteria—a finding that is particularly concerning given that Ecuador represents the westernmost Amazonian range limit for both species, where narrow, swift rivers and limited floodplain habitats naturally constrain carrying capacity. The sole exception, a composite estimate of 1.13 ind/km^2^ for *Sotalia* from the Ramsar CLY site in 2006 (Gómez-Salazar et al., 2012), carries uncertainty substantially above the recommended threshold for reliable density estimation (CV > 0.2; Buckland et al., 2001), with CV = 1.4, and aggregates data from multiple rivers, precluding direct comparison with recent river-specific surveys. Placing these findings in a broader Amazonian context reveals a density gradient across the basin. The highest densities reported anywhere in the Amazon for *I. geoffrensis* occur in the central Brazilian Amazon, particularly in productive white-water rivers such as the lower Purus, where Paschoalini et al. (2021) documented 14.5 ind/km^2^, with exceptional concentrations reaching 64 ind/km^2^ at confluences within the Mamirauá Sustainable Development Reserve. Similarly, *S. fluviatilis* reaches its maximum abundance in the same region (17.14 ind/km^2^ in the lower Purus), with peak values of 28.14 ind/km^2^ recorded at confluences of the Amazon River in Colombia (Gómez-Salazar et al., 2012). In contrast, the Orinoco basin exhibits substantially lower densities (0.9–1.5 ind/km^2^ for *Inia*), with values as low as 0.57 ind/km^2^ in the main channel of the Meta River (Paschoalini et al., 2021). Ecuadorian densities align with the lower end of this Amazon-wide gradient (Paschoalini et al., 2021): our estimates for *I. geoffrensis* (0.33–7.61 ind/km^2^) overlap with Orinoco basin values (0.57–1.5 ind/km^2^; Paschoalini et al., 2021) but are substantially lower than central Amazonian benchmarks, where densities can reach 14.5 ind/km^2^ in productive white-water rivers such as the lower Purus. Even the highest Ecuadorian value (7.61 ind/km^2^ in Lagartococha, 2022) falls near the lower range of values reported for the Brazilian Amazon (*e.g.*, 14.5–64 ind/km^2^ at confluences). For *S. fluviatilis*, our reported densities (0.05–0.45 ind/km^2^) are among the lowest documented across the species’ entire distribution, an order of magnitude below the 17.14 ind/km^2^ recorded in the central Amazon (Paschoalini et al., 2021). This contrast highlights the acute vulnerability of populations at this western distributional edge.

### Natural constraints and cumulative anthropogenic pressures

This density gradient reflects the interplay of natural biogeographic factors and cumulative anthropogenic pressures. The productivity of central Amazonian white-water rivers, which is typically 2–3 times higher than that of western Amazonian black-water systems (Junk, Bayley & Sparks, 1989), supports larger dolphin populations (Martin & Da Silva, 2004; Gómez-Salazar et al., 2012). Conversely, the Andean-Amazon transition zone in Ecuador is characterized by narrower, swifter rivers with limited floodplain development, inherently constraining carrying capacity (Freshwater Ecoregions of the World, 2020; Bernal et al., 2012). This geomorphological context is exemplified by the Napo River, which descends rapidly from 263 m to 167 m over just 235 km, creating a high-energy, unstable environment with drastic water-level fluctuations, high sediment loads, powerful currents, and minimal floodplain development (Sioli, 2012; Latrubesse, Stevaux & Sinha, 2005). Such conditions differ fundamentally from the stable lowland basins further east and lack the complex, slow-water confluences and floodplain lakes that constitute preferred habitats for both dolphin species in the central Amazon (Martin & Da Silva, 2004; Aliaga-Rossel & McGuire, 2010). Superimposed on these natural constraints, Ecuadorian populations face intensifying anthropogenic threats, including mining pollution, oil extraction, and habitat fragmentation—pressures also documented in the Orinoco and portions of the Peruvian Amazon (RAISG, 2020; Campbell et al., 2022). Quantitative evidence increasingly links such threats to measurable population declines across the basin. Mercury contamination from artisanal gold mining, for instance, has been documented in fish species that constitute dolphin prey bases in the Napo and Pastaza watersheds (Echevarría et al., 2024). Although systematic studies of pollutant load in Amazon River dolphins themselves remain limited, the bioaccumulative nature of heavy metals suggests that populations inhabiting white-water rivers receiving Andean sediment loads may face chronic, sublethal health effects that compound over time. In Ecuador, the convergence of anthropogenic threats and natural habitat constraints at the western range margin likely creates synergistic effects that increase population vulnerability.

### Population fluctuations, spatial patterns, and conservation priorities

Between 2019 and 2024, *I. geoffrensis* in Lagartococha (within the protected Ramsar CLY site) showed marked inter-annual fluctuations: 6.53 ind/km^2^ (2019), 3.07 (2021), 7.61 (2022), and 3.01 (2024). Overlapping confidence intervals for encounter rates between 2019 (0.11–1.61 ind/km) and 2024 (0.49–0.97 ind/km) indicate that these differences are not statistically significant; therefore, a consistent declining trend is not demonstrated. Given methodological differences in spatial coverage across surveys, the precise magnitude and direction of change remain uncertain. Nonetheless, the repeated occurrence of low densities (*e.g.*, 3.01 ind/km^2^ in 2024)—which fall below central Amazonian benchmarks and meet Ecuador’s Critically Endangered thresholds for small population size and restricted range (Tirira, 2021)—underscores the species’ vulnerability. For a slow-reproducing apex predator at its western range limit, even fluctuations with recurring low values represent a conservation concern. Spatial patterns further support this interpretation: the lowest densities of *S. fluviatilis* in Ecuadorian rivers (0.05–0.45 ind/km^2^) coincide geographically with areas of intensive mining activity in southern basins (RAISG, 2020). While establishing direct causality requires targeted investigation, this spatial correspondence raises concern about potential causal links that mining-related habitat degradation may be contributing to the species’ extremely low abundance at its western range limit. The recent confirmation of *I. geoffrensis* in the Morona River (2.90 ind/km^2^ in 2024) expands the species’ known distribution and demonstrates that southern basins are not uniformly depauperate. However, this finding also underscores the urgency of extending standardized surveys to all potentially suitable habitats, as undocumented populations may exist in remote river systems facing imminent threats from extractive activities. Taken together, these findings underscore that western Amazonian populations face compounding threats from natural habitat constraints (Vidal et al., 1997) and expanding anthropogenic pressures. The convergence of inherently limited carrying capacity with accelerating human impacts places Ecuador’s river dolphins in a precariously vulnerable position. Demographic parameter estimation and population trend analysis must therefore become a coordinated national priority, requiring sustained monitoring efforts across Ecuador’s Amazonian watersheds to inform effective conservation strategies before local extirpations occur.

### Updated distributional records

Our study updates the known distribution of *Inia geoffrensis* in Ecuador, confirming its presence in the Morona River basin—a system previously unreported for the species (Utreras, Suárez & Jalil, 2010). This finding aligns with recent work by Aguilar & Goucher (2024), who first documented *I. geoffrensis* in the Morona River, subsequently verified through standardized surveys by the WWF monitoring program. These records contrast with earlier IUCN assessments, which noted the absence of the species in the Morona River (Best & Da Silva, 1993; Utreras, Trujillo & Usma, 2013). Additionally, we documented *I. geoffrensis* in several tributaries of the Morona (Wichimi, Makuma, Cangaime, Cushimi, Mangosiza) and the lower Santiago River, further extending its known range within the Ecuadorian Amazon. While the elevation range (<260 m) remains consistent with historical records, these new occurrences underscore the species’ adaptability to diverse riverine habitats, including systems previously considered uninhabited.

Our findings align with previous reports (Utreras, Suárez & Jalil, 2010; Utreras, Trujillo & Usma, 2013; Gómez-Salazar et al., 2010) on the known distribution of *Sotalia fluviatilis*, with no new rivers identified for this species. Northern populations demonstrate greater persistence in confluences than in other habitat types, where mixing waters create critical hydrological nodes that appear to seasonally concentrate prey resources and dolphin activity—a pattern consistent with optimal foraging strategies observed in other river dolphin species (Gómez-Salazar et al., 2012). While the species remains most frequently observed in these confluence zones, we document sporadic occurrences in the lower Tiputini, Yasuní, and southern tributaries (Pastaza and Morona basins), revealing a patchy distribution with 80% of sightings occurring in only three of the thirteen occupied rivers. It is important to note that our *Sotalia* estimates are restricted to northern rivers, as this was the only region with systematically collected survey data using standardized protocols. While occasional sightings have been reported in southern rivers (Zapata-Ríos & Utreras, 2004), we excluded opportunistic records from our quantitative analyses to ensure methodological consistency—a geographic limitation that underscores the urgent need for expanded survey effort across the species’ potential range in Ecuador, particularly in southern basins where data remain scarce. This fragmented occurrence pattern likely reflects both natural habitat constraints and mounting anthropogenic pressures, especially in southern basins where sediment loading from Andean foothill mining operations has degraded aquatic habitats (RAISG, 2020). Of particular concern is the species’ apparent absence in recent systematic monitoring of the Pastaza River mainstem, despite known connectivity to Peruvian populations where *S. fluviatilis* remains present (Da Silva et al., 2020). This disjunction suggests either: (1) undocumented ecological barriers to dispersal, potentially related to altered water chemistry or flow regimes; or (2) localized extirpation due to cumulative anthropogenic impacts—a hypothesis requiring urgent investigation given the species’ Critically Endangered status in Ecuador (Tirira, 2021). These findings reinforce Ecuador’s role as the western range limit for *S. fluviatilis* in the Amazon basin, mirroring the marginal distribution observed in *Inia geoffrensis*.

### Habitat-specific vulnerabilities

Group sizes varied significantly by habitat type, with mean groups at confluences (2.62 ± 0.98) being more than twice as large as those around river islands (1.14 ± 0.38). (Table 5, Fig. 6). This pattern suggests resource concentration in hydrologically dynamic zones (Martin & Da Silva, 2004). These patterns likely reflect underlying habitat preferences, as river dolphin group sizes are known to vary substantially across ecosystem types in response to prey availability and hydrological dynamics throughout the Amazon basin (Gómez-Salazar et al., 2012). The concentration of larger groups in confluences, zones of mixing waters and elevated fish abundance, supports optimal foraging strategies observed across the species’ range. This preference is quantitatively reinforced by Rai et al. (2023), who demonstrated that all three obligate freshwater dolphin species show peak occurrence within 250–1,250 m of confluences, where stable flows and fish aggregation create critical foraging nodes. However, confluences face greater threats from shipping traffic than other habitat types, potentially disrupting social structures (*e.g.*, Aguarico River; Castello & Macedo, 2016; Campbell et al., 2022). The vulnerability of these habitats is further heightened by hydrologic alterations from mining and oil extraction activities, which degrade water quality, increase sediment loads, and alter river dynamics, pressures increasingly evident in Ecuadorian basins (RAISG, 2020; Campbell et al., 2022). The scarcity of *S. fluviatilis* records in southern basins (Curaray/Pastaza) correlates with elevated sediment pollution from Andean foothill mining (RAISG, 2020) and to a lesser extent to dolphin hunting activities for illegal trafficking (MAATE, 2025), exacerbating its naturally patchy distribution (Zapata-Ríos & Utreras, 2004).

### Methodological and conservation implications

Persistent data gaps (*e.g.*, missing confidence intervals for five of 12 historical surveys), particularly for *Sotalia fluviatilis*, underscore the limitations of non-standardized historical surveys (Zapata-Ríos & Utreras, 2004; MAATE, 2025). Our integrated approach, combining National Biodiversity Database (BNDB) occurrence records with standardized SARDI protocols, addresses these gaps and establishes a quantitative baseline for future monitoring. Post-2019 standardized data reveal inter-annual differences ranging from 54% to 86% across the three Ramsar CLY rivers (*e.g.*, *I. geoffrensis* in Lagartococha: 6.53 ind/km^2^ in 2019 *vs.* 3.01 ind/km^2^ in 2024); however, overlapping confidence intervals indicate that these differences are not statistically significant, so a consistent declining trend is not demonstrated. The primary value of these observations is to highlight the need for sustained, standardized monitoring to distinguish natural fluctuations from potential anthropogenic impacts. Additionally, *S. fluviatilis* densities (0.05–0.45 ind/km^2^) are among the lowest documented across its entire distribution (Gómez-Salazar et al., 2010), reinforcing the urgency of expanded survey effort. Nevertheless, the spatial patterns in encounter rates presented here must be interpreted with consideration of underlying methodological factors: first, methodological heterogeneity across surveys, particularly the absence of confidence intervals in five datasets (predominantly from pre-2019 studies), limits the precision of historical comparisons; second, variable spatial units, including aggregated counts from multiple river systems (*e.g.*, Ramsar CLY site), complicate fine-scale interpretation of population trends; and third, potential transboundary sampling bias in the Napo River, where binational surveys included both Ecuadorian and Peruvian waters, may affect encounter rate comparability with purely national datasets. For *S. fluviatilis* specifically, data quality further constrains interpretation: most historical surveys lacked coefficients of variation or confidence intervals, and even where reported (*e.g.*, Aguarico 2019, ±0.04), variability remained high. These limitations reflect both the species’ low detectability (confirmed by zero sightings in 40% of transects) and the urgent need for increased survey effort (at least 60 sightings per region for robust CV <0.2) across its Ecuadorian range. Together, these considerations underscore the critical importance of standardized, replicated survey protocols—such as those developed by SARDI—for robust regional population assessment (Gómez-Salazar et al., 2012), and highlight the urgent need for systematic, long-term monitoring using unified methodologies to track populations effectively and inform targeted conservation actions in this understudied western Amazonian frontier.

### Future directions for research and conservation in ecuador

#### Research and policy directions

The 54–86% density reductions observed in this study (though not all statistically significant) underscore the urgency of targeted research and conservation actions. Through the newly formed National River Dolphin Research and Conservation Group, a member of SARDI, Ecuador is positioned to implement its National Action Plan (MAATE, 2025) through five priority actions: (1) Systematic population assessments across all occupied rivers using standardized monitoring, addressing current spatial and temporal data gaps to establish robust baselines for trend detection; (2) movement ecology studies utilizing satellite telemetry and acoustic monitoring to identify critical habitats, seasonal movement patterns, and transboundary corridors essential for informing protected area design and binational agreements with Peru and Colombia; (3) toxicological assessments quantifying mercury and other heavy metal concentrations in dolphin tissues, building on evidence of contaminated prey bases (Echevarría et al., 2024) to evaluate health risks and identify pollution hotspots requiring mitigation; (4) advocacy for international recognition under CMS and CITES, strengthening legal frameworks to ensure dolphin habitat is considered in environmental impact assessments for infrastructure projects (dams, oil extraction, mining); and (5) establishment of a national conservation site network, securing protected status for rivers that support viable populations but currently lack formal protection (*e.g.*, Curaray, Morona). Together, these actions will transform current knowledge gaps (*e.g.*, *Sotalia* status in southern basins, mercury bioaccumulation rates, transboundary movement patterns) into actionable conservation strategies, safeguarding Ecuador’s critically endangered river dolphins at their western Amazonian range limit.

### Community-led and sustainable initiatives

Sustainable conservation outcomes depend on robust collaboration with riverside communities (Trujillo et al., 2010). Priority actions include: (1) Participatory monitoring networks that integrate Traditional Ecological Knowledge (TEK) to generate annual presence/absence data while building local capacity for stewardship. TEK has proven instrumental in identifying critical habitats, documenting emerging threats (*e.g.*, dolphin carcass use as bait in fisheries), and informing bycatch mitigation strategies based on fishers’ knowledge of dolphin-fisheries interactions (Iriarte & Marmontel, 2013); (2) environmental education programs promoting sustainable fisheries to reduce bycatch and prey depletion, key threats from artisanal fishing, subsistence hunting, and small-scale gold mining that currently provide essential income but carry environmental costs; and (3) dolphin-watching ecotourism initiatives offering non-extractive economic alternatives that transform human-dolphin interactions from competition to active stewardship, positioning dolphins as economic assets rather than perceived competitors for fish. Communities that traditionally coexist with dolphins often possess sustainable practices; engaging local knowledge holders in designing alternative livelihoods fosters long-term stewardship while reducing reliance on extractive activities. This transition to community-managed ecotourism can generate comparable or greater income than extractive activities while reducing anthropogenic pressure on critical habitats. The National Action Plan’s goals (MAATE, 2025) should be operationalized through community workshops, school curricula, and multimedia campaigns in local languages, ensuring strategies reflect both scientific priorities and community needs. This integrated approach, combining cutting-edge research, proactive policy, and deep community engagement, offers the most promising path to safeguard Ecuador’s critically endangered river dolphins and the ecological integrity of their freshwater habitats.

### Study limitations and data considerations

Several limitations should be considered when interpreting our findings. First, sample sizes ranged from three to 27 sightings per river, below the recommended minimum of 60 sightings for a low coefficient of variation (CV < 0.2; Buckland et al., 2001). For *Sotalia fluviatilis*, low sighting frequencies (often 1–6 individuals per survey) resulted in high CVs and precluded robust density estimation for many rivers. Reliable density estimates typically require > 15 transects and ≥60 sightings to achieve CV < 0.2 (Buckland et al., 2001; Gómez-Salazar et al., 2012). Although our survey design included >15 transects for most rivers (Table 1), the number of sightings per river ranged from 3 to 27, falling below this threshold in many cases. This limitation is reflected in the high CVs observed, particularly for *Sotalia* and in remote headwater rivers where detection rates were lowest (Table 4). We therefore advise cautious interpretation of estimates with high CVs, as these should be considered indicative rather than definitive.

Second, unequal sampling effort across rivers (12.5 km in Ishpingo to 205 km in Curaray) reflects logistical constraints inherent to remote Amazonian fieldwork. Although our stratified analytical approach partially accounts for this imbalance, rivers with limited effort yield less precise estimates and should be prioritized in future surveys. Third, temporal and seasonal biases may influence comparability across datasets; while we standardized surveys to transitional hydrological periods whenever possible, some historical datasets lacked seasonal metadata, precluding adjustment for flood pulse effects on detectability and habitat use (Martin & Da Silva, 2004; Gómez-Salazar et al., 2012). We also acknowledge that abundance (N), density (D), and encounter rate (ER) are typically linearly related under ideal conditions, but inconsistencies can arise from methodological factors. Encounter rates are raw indices that do not account for detectability, whereas density estimates incorporate detection probabilities and depend on habitat-specific area calculations from satellite imagery, which vary across rivers and hydrological periods. Transboundary or composite datasets (*e.g.*, Napo 2021, Ramsar CLY 2006) introduce further heterogeneity. These factors likely explain observed discrepancies in rivers such as Yasuní, where density remained relatively stable (2.03–4.96 ind/km^2^) while encounter rates declined (0.32 to 0.11 ind/km) (Fig. 4). Such patterns may reflect shifts in habitat use, detectability, or survey timing rather than actual population change. Despite these constraints, the implementation of standardized SARDI protocols since 2019 has improved data quality and inter-survey comparability, providing a robust baseline for future monitoring. Addressing the remaining gaps will require sustained, strategically targeted survey effort across Ecuador’s under-sampled river systems.

## Conclusions

This first standardized, nationwide assessment confirms the critically endangered status of river dolphins at the western limit of their Amazonian range in Ecuador. Both species exhibit densities that are among the lowest documented for any Amazonian River dolphin population, with *Inia geoffrensis* ranging from 0.33 to 7.61 ind/km^2^ and *Sotalia fluviatilis* from 0.05 to 0.45 ind/km^2^ across recently surveyed rivers. The sole exception, a composite estimate of 1.13 ind/km^2^ for *Sotalia* from the Ramsar CLY site in 2006, carries uncertainty far exceeding the acceptable CV threshold of 0.2 (CV = 1.4) and predates the implementation of standardized survey protocols, underscoring the importance of methodological consistency for robust population assessment. For *S. fluviatilis*, recent estimates represent some of the lowest densities documented across the species’ entire distribution, highlighting the acute vulnerability of populations at this western range edge. We update the known distribution of *I. geoffrensis* to 37 rivers, including new standardized survey data from the Morona River (2.90 ind/km^2^ in 2024), while confirming the restricted range of *S. fluviatilis* to 13 rivers in Ecuador’s northern Amazon. These findings establish Ecuador as the definitive western Amazonian boundary for both species and identify Lagartococha as a critical stronghold for *Inia*. However, marked inter-annual fluctuation (3.01–7.61 ind/km^2^) and a 54% difference between the 2019 and 2024 estimates (though not statistically significant) raise conservation concerns regarding population vulnerability.

Above all, this study provides the first standardized baseline for river dolphin abundance in Ecuador, enabling future researchers to detect real trends as anthropogenic pressures intensify. The convergence of naturally constrained habitats at the Andean-Amazon transition zone with intensifying anthropogenic pressures—including mining pollution, oil extraction, and habitat fragmentation—creates a synergistic effect that places Ecuador’s river dolphins in a precariously vulnerable position. The spatial correspondence between the lowest *Sotalia* densities and areas of intensive mining activity in southern basins, while not yet demonstrating causality, warrants urgent investigation. The urgent implementation of Ecuador’s National Action Plan for river dolphin conservation is paramount. Priority actions must include: (1) sustained, standardized monitoring using SARDI protocols across all occupied rivers to track population trends with consistent spatial and temporal coverage; (2) targeted research on specific threats, particularly mercury contamination, bycatch, and habitat fragmentation; (3) transboundary coordination with Peru and Colombia to maintain connectivity across the western Amazon; and (4) expansion of survey effort to under-sampled southern basins, where recent Morona River findings demonstrate that locally suitable habitats may exist but remain poorly characterized. Without immediate, coordinated action focused on protecting key habitats and engaging local communities, local extirpations of these apex predators are likely, with cascading consequences for freshwater ecosystem integrity.

##  Supplemental Information

10.7717/peerj.21586/supp-1 Supplemental Information 1 Field data collected by the authors The complete, raw field data from boat-based visual surveys conducted for the conservation status assessment of river dolphins (*Inia geoffrensis* and *Sotalia fluviatilis*) in the Ecuadorian Amazon. Data were collected across multiple rivers and lagoons during both raising and falling water seasons between 2019 and 2024.

10.7717/peerj.21586/supp-2 Supplemental Information 2 Consolidated and static dataset of georeferenced occurrence records for the river dolphin species *Inia geoffrensis* and *Sotalia fluviatilis* within Ecuador These historical records, which form the basis for the Distribution Range Analysis in this study, include the following fields for each record: scientificName, recordedBy, River, decimalLatitude, decimalLongitude, and references.

## Additional Information and Declarations

### Data Availability

The following information was supplied regarding data availability: The data is available in the Supplemental Files. The data is available at the National Biodiversity Database of Ecuador (BNDB): https://bndb.sisbioecuador.bio/bndb/collections/misc/collprofiles.php?collid=27.
